# A 3′ UTR SNP in COL18A1 Is Associated with Susceptibility to HBV Related Hepatocellular Carcinoma in Chinese: Three Independent Case-Control Studies

**DOI:** 10.1371/journal.pone.0033855

**Published:** 2012-03-26

**Authors:** Xiaopan Wu, Jia Wu, Zhenhui Xin, Huifen Wang, Xilin Zhu, Liping Pan, Zhuo Li, Hui Li, Ying Liu

**Affiliations:** 1 National Laboratory of Medical Molecular Biology, Institute of Basic Medical Sciences, Chinese Academy of Medical Sciences; School of Basic Medicine, Peking Union Medical College, Beijing, People's Republic of China; 2 Liver Failure Treatment and Research Center, The 302 Hospital of the People's Liberation Army, Beijing, People's Republic of China; 3 Department of Infectious Disease, Affiliated Youan Hospital, Capital University of Medical Science, Beijing, People's Republic of China; 4 Department of Epidemiology, Institute of Basic Medical Sciences, Chinese Academy of Medical Sciences; School of Basic Medicine, Peking Union Medical College, Beijing, People's Republic of China; Broad Institute of Massachusetts Institute of Technology and Harvard University, United States of America

## Abstract

**Background:**

Accumulated evidences indicate that single nucleotide polymorphisms (SNP) in angiogenesis and tumorigenesis related genes are associated with risk of Hepatocellular carcinoma (HCC). COL18A1 encodes the precursor of endostatin, which is a broad-spectrum angiogenesis inhibitor, and we speculate that SNPs in COL18A1 may be associated with susceptibility to HCC.

**Methods and Findings:**

We carried out a 2-stage association study in 3 independent case-control groups in a total of 1067 chronic hepatitis B (CHB) patients and 808 hepatitis B virus (HBV) related HCC patients in Han Chinese. Four SNPs which can represent all potential functional SNPs with MAF>0.1 recorded in HapMap database were genotyped using TaqMan methods. Levels of total COL18A1 mRNA were also examined using quantitative real-time RT-PCR. We found that rs7499 located in 3′-UTR to be strongly associated with HBV related HCC (P_combined_ = 0.0000005, OR = 0.72, 95%CI = 0.63–0.82). COL18A1 mRNA expression was significantly decreased as the disease progressed (P = 0.000026).

**Conclusion:**

These findings indicate that COL18A1 rs7499 may contribute to the risk of HCC in Han Chinese.

## Introduction

Hepatocellular carcinoma (HCC) is one of the most common malignancy and ranks fifth in men and eighth in women among causes of cancer mortality worldwide. It is estimated that about 564,000 new cases of HCC are reported throughout the world each year [1]. Eastern Asia is the geographic area at highest risk of HCC [1]. The cause of HCC is a complex interplay between multiple genetic and environmental factors [2]. Hepatitis B virus (HBV) and hepatitis C virus (HCV) are the main viral factors of HCC, while in China, HBV related HCC is the most frequent. On the other hand, accumulated evidences in molecular genetics indicate that single nucleotide polymorphisms (SNP) in immune response, angiogenesis and tumorigenesis related genes are associated with susceptibility to HCC [3], [4], [5], [6]. Recent progress in genome-wide association study (GWAS) also have identified new susceptibility loci for HCC [7], [8].

Collagen, type XVIII, alpha 1 (COL18A1) gene locates at 21q22.3. It has 42 exons, and encodes a protein of 1336aa. This protein is the precursor of endostatin, which is a 20-kDa protein derived from carboxy-terminal proteolytic fragment of collagen XVIII [9]. Endostatin is a broad-spectrum angiogenesis inhibitor and interferes with growth factors such as VEGF [10], and has the potency to inhibit neovascularization and tumor growth [11]. Angiogenesis plays an important role in tumorigenesis. Tumours secrete a number of angiogenic growth factors such as VEGF. Furthermore, the expression of endogenous inhibitors, for instance endostatin and angiostatin, are downregulated [12]. Studies have reported that endostatin expression was significantly stronger in adjacent nontumor tissues than that in tumors in HCC specimen [13], and effectively inhibit the growth of HCC [14].

According to the above evidence, we speculate that SNPs in COL18A1 may be associated with susceptibility to HCC. We used a candidate gene strategy and carried out a 2-stage association study to confirm this hypothesis.

## Materials and Methods

### Subjects

The subjects enrolled in the present study included 3 independent case-control groups in a total of 1067 chronic hepatitis B (CHB) patients and 808 HBV related HCC patients.

The first group consisted of 169 HCC patients and 203 CHB patients enrolled from the 302 Hospital of the People's Liberation Army (Beijing) from Jan 2008 to Nov 2009. The second group consisted of 326 HCC patients and 522 CHB patients enrolled from Beijing Youan Hospital (Beijing) from Oct 2005 to Jul 2010. The third group consisted of 313 HCC patients and 342 CHB patients enrolled from Guangxi Cancer Hospital (Nanning, Guangxi) from May 2005 to Jan 2008.

Controls were CHB patients whose serum levels of alanine aminotransferase (ALT) and aspartate aminotransferase (AST) were continuously >40 IU/L; they were HBsAg seropositive and HBeAg seropositive for 6 months; their serum HBV DNA >2,000 copies/mL and confirmed by liver ultrasonography.

Cases were pathologically HCC patients, pathologically confirmed, and proved not to have other cancers. They were also confirmed by liver ultrasonography and/or computed tomography.

Subjects were considered smokers if they smoked up to 6 months before the date of cancer diagnosis for HCC cases or the date of interview for CHB controls. An alcohol drinker was defined as someone who consumed alcohol at least once per week for at least 6 months.

The subjects were excluded if: (1) there was evidence of past or current infection with other hepatitis viruses or hepatitis not caused by HBV; (2) they were not of Han ethnicity. The main features of the subjects included are summarized in Table 1 and Table S1. The study was carried out in accordance with the guidelines of the Helsinki Declaration after obtaining written informed consent from all the subjects and was approved by the ethics committee of the Institute of Basic Medical Sciences, Chinese Academy of Medical Sciences.

**Table 1:** Clinical features of the subjects included in the study.

		302_Beijing			Youan_Beijing			Guangxi	
	HCC	CHB	P	HCC	CHB	P	HCC	CHB	P
Number	169	203		326	522		313	342	
Age, *y*	50 (42, 55)	49 (38, 57)	0.37^a^	52 (45, 58)	40 (34, 49)	<0.001^a^	43 (37, 51)	41 (35, 52)	0.10^a^
Gender (male/female)	143/26	158/45	0.10^b^	270/56	379/143	<0.001^b^	280/33	283/59	0.01^b^
Smoking (Yes/No)	84/85	64/139	<0.001^b^	135/159	103/378	<0.001^b^	128/185	141/201	0.93^b^
Drinking (Yes/No)	93/76	71/132	<0.001^b^	141/153	113/366	<0.001^b^	95/218	97/245	0.58^b^
Family history of HBV (Yes/No)	98/71	133/70	0.14^b^	141/172	333/169	<0.001^b^			
Family history of HCC (Yes/No)							52/261	16/326	<0.001^b^
HbeAg (+/−)	65/104	131/72	<0.001^b^	128/198	356/166	<0.001^b^			
ALT (IU/L)	218 (67, 227)	227 (113, 268)	<0.001^a^	129 (109, 142)	131 (122, 143)	0.01^a^			
AST (IU/L)	158 (72, 230)	160 (96, 245)	0.03^a^	124 (108, 165)	125 (109, 162)	0.88^a^			
TBil (µmol/L)	36 (19, 49)	34 (21, 50)	0.08^a^	49 (28, 61)	39 (22, 54)	<0.001a			
DBil (µmol/L)	28 (15, 35)	29 (15, 36)	0.89^a^	33 (16, 50)	27 (11, 35)	<0.001^a^			
Log HBV-DNA (copy/mL)	5.9 (4.6, 6.2)	6.7 (5.5, 7.2)	<0.001^a^	5.4 (4.6, 6.0)	5.6 (4.7, 6.1)	<0.001^a^			

Age, ALT, AST, TBil, DBil, and HBV-DNA as median (25-percentile, 75-percentile).

HCC: hepatocellular carcinoma.

CHB: chronic hepatitis B.

^*a*^Mann-Whitney U test.

^*b*^Chi-square test.

### SNP selection and genotyping

Genomic DNA was extracted from peripheral blood by using a salting-out protocol [15]. Using the HapMap database (HapMap Data Rel 24/phase II Nov 08, on NCBI B36 assembly, dbSNP b126), potential functional SNPs (SNPs in promoter region and mRNA sequence) with minor allele frequency (MAF) of greater than 0.10 for the Han Chinese Beijing population were selected from the entire gene region from approximately 4000 bp upstream of the transcription start site to 2000 bp downstream of the 3′untranslated region (3′UTR). Five SNPs were found, namely rs2183589 (promoter), rs2230687, rs2230688, rs11702425 (synonymous coding) and rs7499 (3′UTR). Among these SNPs, rs2230687 and rs2230688 were in complete linkage disequilibrium (LD) according to HapMap data, so we chose rs2230688 as a representative. The 4 selected SNPs were genotyped by TaqMan method, with probes synthesized by Sangon BioTech Co Ltd (Shanghai, China). Primers and TaqMan probes used are listed in Table S2. All the samples were successfully genotyped. For genotyping quality control, 5% samples were randomly selected and directly sequenced, and we obtained 100% identical results.

### Quantitative Analysis of Gene Expression

Total RNA was extracted from the peripheral blood by RNAprep pure Blood Kit (TianGen, Beijing, China). cDNA was synthesize in a 20 uL reaction volume containing Oligo (dT)18 primers (MBI Fermentas, Lithuania), and RevertAid™ H minus M-MuLV reverse transcriptase (MBI Fermentas, Lithuania). Expression of COL18A1 mRNA was measured by TaqMan relative quantitative analysis using the Bio-Rad iQ5 Real-Time PCR Detection system (Bio-Rad, Hercules, CA). Glyceraldehyde-3-phosphate dehydrogenase (GAPDH) was used as an internal control gene. The primers and probe used for amplification of COL18A1 and GAPDH cDNA samples are listed in Table 2. The amplifications of COL18A1 and GAPDH cDNA of each sample were performed in the same 96-well PCR plate. Each experiment was performed in triplicate assay. The comparative Ct (2^−ΔΔCt^) method was employed to quantify COL18A1 expression as described previously [16].

**Table 2:** Genotype distributions of 4 SNPs in COL18A1 gene of the 302_Beijing samples.

			Allele, n (%)			Genotype, n (%)		Cochran Armitage trend test	logistic regression#
	1/2	1	2	P/OR (95% CI)	11	12	22	P	P/OR (95% CI)
rs2183589	C/T								
HCC (n = 169)		306 (90.5%)	32 (9.5%)	0.18	139 (82.2%)	28 (16.6%)	2 (1.2%)	0.18	0.20
CHB (n = 203)		355 (87.4%)	51 (12.6%)	1.37 (0.86–2.19)	155 (76.4%)	45 (22.2%)	3 (1.5%)		1.37 (0.85–2.22)
rs2230688	G/T								
HCC (n = 169)		223 (66.0%)	115 (34.0%)	0.43	72 (42.6%)	79 (46.7%)	18 (10.7%)	0.43	0.41
CHB (n = 203)		279 (68.7%)	127 (31.3%)	0.88 (0.65–1.20)	99 (48.8%)	81 (39.9%)	23 (11.3%)		0.88 (0.64–1.20)
rs11702425	T/C								
HCC (n = 169)		262 (77.5%)	76 (22.5%)	0.98	104 (61.5%)	54 (32.0%)	11 (6.5%)	0.98	0.73
CHB (n = 203)		315 (77.6%)	91 (22.4%)	0.99 (0.70–1.41)	127 (62.6%)	61 (30.0%)	15 (7.4%)		0.94 (0.67–1.32)
rs7499	C/T								
HCC (n = 169)		153 (45.3%)	185 (54.7%)	0.006	29 (17.2%)	95 (56.2%)	45 (26.6%)	0.005	0.009
CHB (n = 203)		225 (55.4%)	181 (44.6%)	0.67 (0.50–0.89)	63 (31.0%)	99 (48.8%)	41 (20.2%)		0.66 (0.48–0.90)

# P values were adjusted for age, gender, smoking and drinking by binary logistic regression.

### Statistical analysis

By using the χ2 test, we tested whether the genotype distributions for the studied SNP were in the Hardy-Weinberg equilibrium (HWE). We used 2×2 or 2×3 contingency tables for comparing allele and genotype frequencies between groups. Tests for differences of quantitative traits and COL18A1 mRNA expression between different groups were performed using the Mann-Whitney U test or Kruskal-Wallis Test for traits with nonnormal distributions, or ANOVA for normally distributed traits. P<0.05 was the criterion for statistical significance. All statistical analyses were performed using the Statistical Package for the Social Sciences (SPSS), version 12.0. We obtained estimates of LD values (r2, D′) and the haplotype estimation using the SHEsis online software [17].

## Results

We first conducted genotyping experiments for the 4 COL18A1 polymorphisms in the 302_Beijing group of samples. Genotype distributions of the studied SNPs were in HWE in both cases and controls. The genotype distributions and allelic frequencies of COL18A1 polymorphisms in CHB and HCC patients were represented in Table 2. The frequency of C allele of rs7499 was 45.3% in HCC patients vs. 55.4% in CHB patients (P = 0.006, OR = 0.67, 95%CI = 0.50–0.89). The Cochran-Armitage trend test (assuming an additive model for C allele) revealed an allele dose-dependent association of rs7499 with the HCC (P = 0.005), with increased OR of 2.08 and 2.38 for CT and TT genotypes, respectively. We then used binary logistic regression to adjust for confounding factors as age, gender, smoking and drinking under additive model, and the result showed that rs7499 was still independently associated with HCC (P = 0.009, OR = 0.66, 95%CI = 0.48–0.90). The 3 other SNPs, however, were not associated with HCC under any model. We analyzed the degree of LD for these 4 SNPs, and found there was no apparent LD (D′≤0.291, r^2^≤0.017). Table 3 shows 5 common haplotypes constructed by these 4 SNPs. The C-T-T-T haplotype was associated with HCC most significantly (P = 0.0002). Notably, the C-T-T-C haplotype, which was different with C-T-T-T haplotype only at the rs7499 locus, was also associated with HCC (P = 0.01), but with opposite tendency. These results were in accordance with the single locus analysis, suggesting that rs7499 was a principal genetic factor in COL18A1.

**Table 3:** Common haplotypes constructed with SNPs rs2183589, rs2230688, rs11702425 and rs7499 in COL18A1 gene of the 302_Beijing samples.

Haplotypes	HCC (n = 169)	CHB (n = 203)	P	OR (95%CI)
C-G-C-T	42.84 (12.7%)	24.33 (6.0%)	0.003	2.20 (1.31–3.71)
C-G-T-C	77.97 (23.1%)	113.98 (28.1%)	0.06	0.73 (0.52–1.02)
C-G-T-T	63.69 (18.8%)	76.35 (18.8%)	0.82	0.96 (0.66–1.39)
C-T-T-C	24.70 (7.3%)	51.37 (12.7%)	0.01	0.52 (0.31–0.86)
C-T-T-T	68.67 (20.3%)	40.61 (10.0%)	0.0002	2.22 (1.46–3.39)
others	60.13 (17.8%)	99.36 (24.5%)		

We next replicated genotyping of rs7499 in 2 independent case-control samples from Beijing and Guangxi respectively. The result was presented in Table 4. rs7499 was associated with HCC significantly both with the same tendency as the first group of samples. Except that in group 2, when using binary logistic regression to adjust age, gender, smoking and drinking, the association was disappeared (P = 0.29). We combined the 3 groups together, in a total of 1067 CHB patients and 808 HCC patients, the frequency of C allele of rs7499 was 42.5% in HCC patients vs. 50.8% in CHB patients (P = 0.0000005, OR = 0.72, 95%CI = 0.63–0.82). The Cochran-Armitage trend test and binary logistic regression also indicated rs7499 to be strongly associated with HCC (P = 0.0000004, P = 0.00001, respectively).

**Table 4:** Association results for rs7499 in replication studies.

		Allele, n (%)			Genotype, n (%)		Cochran Armitage trend test	logistic regression#
	C	T	P/OR (95% CI)	CC	CT	TT	P	P/OR (95% CI)
Youan_Beijing								
HCC (n = 326)	297 (45.6%)	355 (54.4%)	0.003	63 (19.3%)	171 (52.5%)	92 (28.2%)	0.003	0.29
CHB (n = 522)	552 (52.9%)	492 (47.1%)	0.75 (0.61–0.91)	151 (28.9%)	250 (47.9%)	121 (23.2%)		0.88 (0.70–1.11)
Guangxi								
HCC (n = 313)	237 (37.9%)	389 (62.1%)	0.01	41 (13.1%)	155 (49.5%)	117 (37.4%)	0.01	0.01
CHB (n = 342)	307 (44.9%)	377 (55.1%)	0.75 (0.60–0.93)	70 (20.5%)	167 (48.8%)	105 (30.7%)		0.75 (0.60–0.94)
Combined*								
HCC (n = 808)	687 (42.5%)	929 (57.5%)	5e-7	133 (16.5%)	421 (52.1%)	254 (31.4%)	4e-7	0.00001
CHB (n = 1067)	1084 (50.8%)	1050 (49.2%)	0.72 (0.63–0.82)	284 (26.6%)	516 (48.4%)	267 (25.0%)		0.73 (0.64–0.84)

# P values were adjusted for age, gender, smoking and drinking by binary logistic regression.

* Combination of 3 case-control samples from 302_Beijing, Youan_Beijing and Guangxi.

We examined the quantitative traits such as ALT, AST, total bilirubin (TBil), direct bilirubin (DBil) and HBV DNA levels with different genotypes among CHB and HCC patients respectively to evaluate the association between genotypes of SNP rs7499 and phenotypes. The results showed that the quantitative traits did not significantly differ with the three genotypes of rs7499 (data not shown).

**Figure 1:**
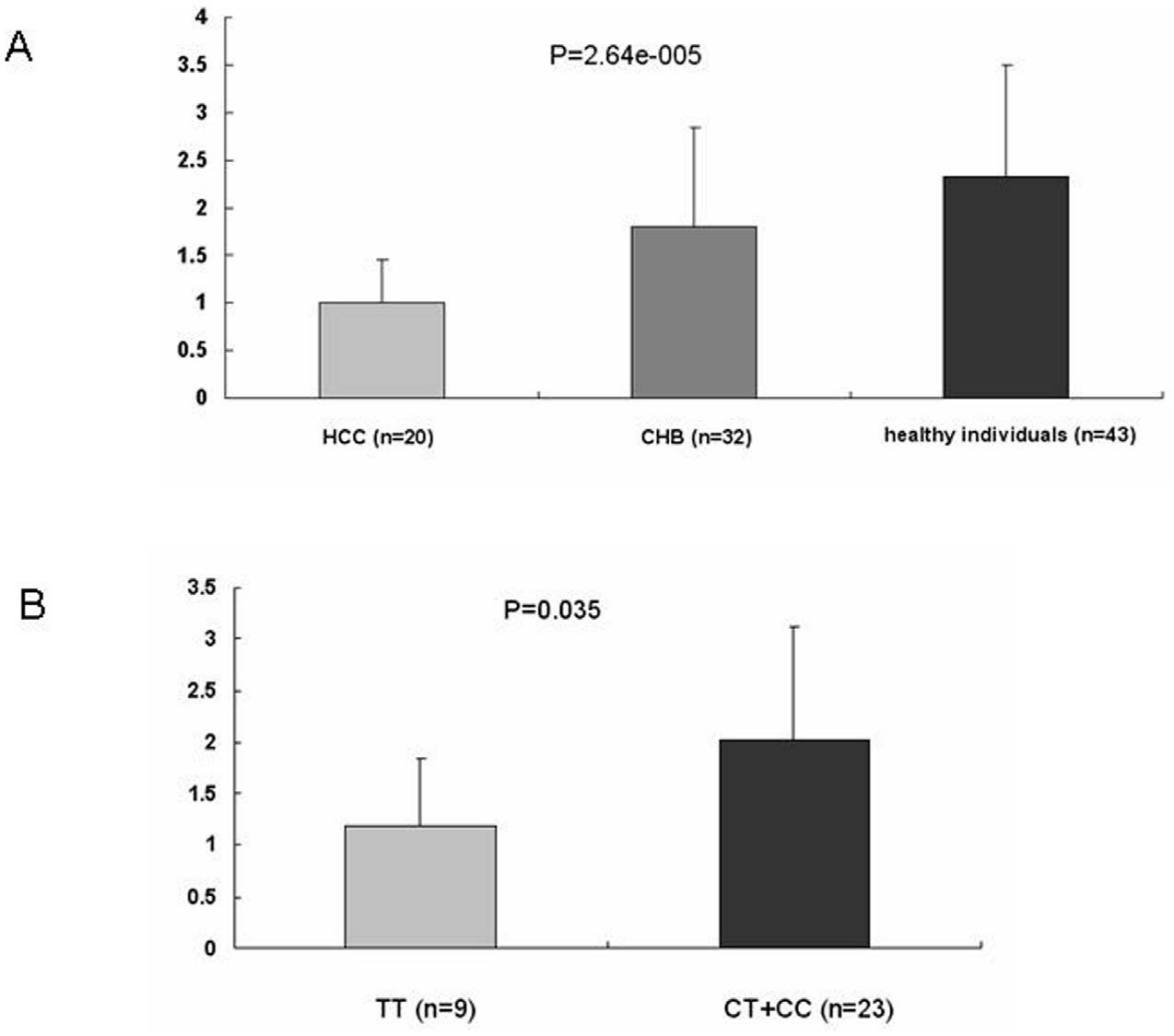
Quantification of COL18A1 mRNA expression by real-time PCR. GAPDH was used as an internal control gene. Final abundance figures were adjusted to yield an arbitrary value of 1 for HCC patients. Each experiment was performed in triplicate assay. Data are means±SD. A. COL18A1 mRNA expression in HCC, CHB, and healthy individuals. B. COL18A1 mRNA expression in CHB patients, among different genotypes of rs7499.

We then examined levels of total COL18A1 mRNA using quantitative real-time RT-PCR. Expression of COL18A1 mRNA levels was measured in RNA from WBCs in 20 HCC patients, 32 CHB patients and 43 HBsAg-negative healthy individuals. Expression levels of COL18A1 mRNA for the three different genotypes of rs7499 were also compared in CHB patients and HBsAg-negative healthy individuals. Final abundance figures were adjusted to yield an arbitrary value of 1 for HCC patients (Figure 1A). The result showed that the HBsAg-negative healthy individuals had 2.33-fold higher COL18A1 mRNA expression than HCC patients and CHB patients had 1.79-fold higher COL18A1 mRNA expression than HCC patients (P = 0.000026). The COL18A1 mRNA level between three different genotypes of rs7499 in healthy individuals had no significant differences (CC: 1.81±0.75; CT: 2.39±1.23; TT: 2.46±1.22; P = 0.60). But in CHB patients, TT carriers had significant lower COL18A1 mRNA level than non-TT carriers (1.18±0.67 vs. 2.03±1.08, P = 0.035), as shown in Figure 1B.

## Discussion

As an anti-angiogenesis gene, COL18A1 plays an important role in tumorigenesis. Studies have reported that SNPs in COL18A1 are associated with numerous cancers, such as breast cancer [18], [19], prostate carcinoma [20], [21], colorectal adenocarcinoma [22] and lung cancer [23]. A non synonymous SNP, D104N, located in the 42th exon of COL18A1, is widely studied [18], [19], [20], [21], [22], [23]. However, the MAF of this SNP is low in HCB population, about 0.047 according to HapMap data, as we selected SNPs with MAF>0.1, the D104N polymorphism was not included in the present study. Further studies are needed to elucidate the role of D104N in HCC.

The distribution of gene polymorphism differed greatly in different ethnicity and region. In the present study, we collected samples from south and north of China, which may represent the population differences. The genotype distribution of rs7499 between Beijing and Guangxi population is different (Table 2 and Table 4); however, the tendency of differences between CHB and HCC groups is similar. The association between rs7499 and HCC has been replicated in both Beijing and Guangxi populations, which indicates that this polymorphism may have a genetic influence in the development of HCC.

rs7499 locates in the 3′-UTR region. After retrieving the NCBI dbSNP (http://www.ncbi.nlm.nih.gov/projects/SNP/snp_gf.cgi), we find that rs7499 is in complete LD with another two 3′-UTR SNPs, i.e. rs8199 and rs7867. SNPs in 3′-UTR region may disrupt or create a microRNA binding site so as to repress translation or destabilize mRNA. The “C” to “T” change of rs7499 may disrupt a binding site for a microRNA hsa-mir-328. The analysis was performed with the program miRBase, available at http://www.mirbase.org/index.shtml [24]. These data indicate that rs7499 may be functional itself, or in LD with other functional SNPs. Several recent works have also genotyped rs7499 and studied its association with myopia [25] and ovarian cancer [26]. Interestingly, in these studies, rs7499 were not associated with diseases, but the distribution patterns of different genotypes of rs7499 were similar in different populations.

We examined the COL18A1 mRNA expression and find that the HBsAg-negative healthy individuals and CHB patients had higher mRNA expression than HCC patients. The role of Endostatin/collagen XVIII expression during HCC was controversial [27], [28], [29]. Evidences suggested that free, soluble endostatin inhibited angiogenesis, whereas immobilized form supported the survival and migration of endothelial cells; so in some cases, endostatin/C18 may promote, instead of abolish, angiogenic processes [30], [31]. The present results suggest that since the expression of angiogenesis factors is elevated during HCC tumorigenesis, it is reasonable that expression of angiogenesis inhibitors such as endostatin/C18 is decreased in HCC patients. We also compare the COL18A1 mRNA expression between three different genotypes of rs7499 in HBsAg-negative healthy individuals and CHB patients. The results in CHB patients showed that TT carriers had significant lower COL18A1 mRNA level than non-TT carriers, but in HBsAg-negative healthy individuals, COL18A1 mRNA level between 3 genotypes did not differed significantly. There is possibility that rs7499 genotypes were not directly associated with COL18A1 mRNA expression. For the gene expression regulation is complicated. The role of endostatin expression during HCC was controversial, in some cases, endostatin may promote, instead of abolish, angiogenic processes. On the other hand, the samples for studying gene expression are small, so there is possibility of false negative. The result should be verified in larger sample set.

Several limitations of the present study need to be addressed. Some clinical features between CHB and HCC patients did not match well. Especially in the Youan_Beijing group of samples, when adjusting age, gender, smoking and drinking, the association between rs7499 and HCC is disappeared. In future studies, clinical features should be well matched. Second, the selection of SNPs in our work is based on database searching. Although the 4 SNPs genotyped in our work can represent all potential functional SNPs with MAF>0.1 recorded in HapMap database, it should be noted that some SNPs not recorded in the database may be omitted; sequencing the whole functional region of COL18A1 is needed in future studies.

In conclusion, we carried out a 2-stage association study and found rs7499 located in 3′-UTR region of COL18A1 gene to be strongly associated with HBV related HCC. Further study in other ethnicities, and the present finding to be confirmed with larger sample set in Han Chinese will be needed to clarify the role of this polymorphism.

## Supporting Information

Table S1 Multivariate Analysis for the Clinical Variables.
(DOC)

Table S2 Primers and Probes Used in TaqMan Genotyping and mRNA Quantification.
(DOC)

## References

[1] Bosch FX, Ribes J, Cleries R, Diaz M (2005). Epidemiology of hepatocellular carcinoma.. Clin Liver Dis.

[2] Chen CJ, Chen DS (2002). Interaction of hepatitis B virus, chemical carcinogen, and genetic susceptibility: multistage hepatocarcinogenesis with multifactorial etiology.. Hepatology.

[3] Clifford RJ, Zhang J, Meerzaman DM, Lyu MS, Hu Y (2010). Genetic variations at loci involved in the immune response are risk factors for hepatocellular carcinoma.. Hepatology.

[4] Deng G, Zhou G, Zhang R, Zhai Y, Zhao W (2008). Regulatory polymorphisms in the promoter of CXCL10 gene and disease progression in male hepatitis B virus carriers.. Gastroenterology.

[5] Kong SY, Park JW, Lee JA, Park JE, Park KW (2007). Association between vascular endothelial growth factor gene polymorphisms and survival in hepatocellular carcinoma patients.. Hepatology.

[6] Long XD, Ma Y, Zhou YF, Ma AM, Fu GH (2010). Polymorphism in xeroderma pigmentosum complementation group C codon 939 and aflatoxin B1-related hepatocellular carcinoma in the Guangxi population.. Hepatology.

[7] Zhang H, Zhai Y, Hu Z, Wu C, Qian J (2010). Genome-wide association study identifies 1p36.22 as a new susceptibility locus for hepatocellular carcinoma in chronic hepatitis B virus carriers.. Nat Genet.

[8] Kumar V, Kato N, Urabe Y, Takahashi A, Muroyama R (2011). Genome-wide association study identifies a susceptibility locus for HCV-induced hepatocellular carcinoma.. Nat Genet.

[9] O'Reilly MS, Boehm T, Shing Y, Fukai N, Vasios G (1997). Endostatin: an endogenous inhibitor of angiogenesis and tumor growth.. Cell.

[10] Coulon S, Heindryckx F, Geerts A, Van Steenkiste C, Colle I (2010). Angiogenesis in chronic liver disease and its complications.. Liver Int.

[11] Boehm T, Folkman J, Browder T, O'Reilly MS (1997). Antiangiogenic therapy of experimental cancer does not induce acquired drug resistance.. Nature.

[12] Dameron KM, Volpert OV, Tainsky MA, Bouck N (1994). Control of angiogenesis in fibroblasts by p53 regulation of thrombospondin-1.. Science.

[13] Hu TH, Huang CC, Wu CL, Lin PR, Liu SY (2005). Increased endostatin/collagen XVIII expression correlates with elevated VEGF level and poor prognosis in hepatocellular carcinoma.. Mod Pathol.

[14] Liu H, Peng CH, Liu YB, Wu YL, Zhao ZM (2005). Inhibitory effect of adeno-associated virus-mediated gene transfer of human endostatin on hepatocellular carcinoma.. World J Gastroenterol.

[15] Miller SA, Dykes DD, Polesky HF (1988). A simple salting out procedure for extracting DNA from human nucleated cells.. Nucleic Acids Res.

[16] Livak KJ, Schmittgen TD (2001). Analysis of relative gene expression data using real-time quantitative PCR and the 2(-Delta Delta C(T)) Method.. Methods.

[17] Shi YY, He L (2005). SHEsis, a powerful software platform for analyses of linkage disequilibrium, haplotype construction, and genetic association at polymorphism loci.. Cell Res.

[18] Balasubramanian SP, Cross SS, Globe J, Cox A, Brown NJ (2007). Endostatin gene variation and protein levels in breast cancer susceptibility and severity.. BMC Cancer.

[19] Lourenco GJ, Cardoso-Filho C, Goncales NS, Shinzato JY, Zeferino LC (2006). A high risk of occurrence of sporadic breast cancer in individuals with the 104NN polymorphism of the COL18A1 gene.. Breast Cancer Res Treat.

[20] Iughetti P, Suzuki O, Godoi PH, Alves VA, Sertie AL (2001). A polymorphism in endostatin, an angiogenesis inhibitor, predisposes for the development of prostatic adenocarcinoma.. Cancer Res.

[21] Mucci LA, Stark JR, Figg WD, Schumacher F, Li H (2009). Polymorphism in endostatin, an angiogenesis inhibitor, and prostate cancer risk and survival: A prospective study.. Int J Cancer.

[22] Nascimento H, Rodrigues Coy CS, Navarro Goes JR, Ferreira Costa F, Passos Lima CS (2004). A polymorphism in the angiogenesis inhibitor, endostatin, in sporadic colorectal adenocarcinoma.. Int J Colorectal Dis.

[23] Zambon L, Honma HN, Lourenco GJ, Saad IA, Mussi RK (2008). A polymorphism in the angiogenesis inhibitor, endostatin, in lung cancer susceptibility.. Lung Cancer.

[24] Griffiths-Jones S, Saini HK, van Dongen S, Enright AJ (2008). miRBase: tools for microRNA genomics.. Nucleic Acids Res.

[25] Yip SP, Leung KH, Fung WY, Ng PW, Sham PC (2011). A DNA pooling-based case-control study of myopia candidate genes COL11A1, COL18A1, FBN1, and PLOD1 in a Chinese population.. Mol Vis.

[26] Permuth-Wey J, Chen Z, Tsai YY, Lin HY, Chen YA (2011). MicroRNA Processing and Binding Site Polymorphisms Are Not Replicated in the Ovarian Cancer Association Consortium.. Cancer Epidemiol Biomarkers Prev.

[27] Yamagata M, Shiratori Y, Dan Y, Shiina S, Takayama T (2000). Serum endostatin levels in patients with hepatocellular carcinoma.. Ann Oncol.

[28] Musso O, Rehn M, Theret N, Turlin B, Bioulac-Sage P (2001). Tumor progression is associated with a significant decrease in the expression of the endostatin precursor collagen XVIII in human hepatocellular carcinomas.. Cancer Res.

[29] Dhar DK, Ono T, Yamanoi A, Soda Y, Yamaguchi E (2002). Serum endostatin predicts tumor vascularity in hepatocellular carcinoma.. Cancer.

[30] Dixelius J, Larsson H, Sasaki T, Holmqvist K, Lu L (2000). Endostatin-induced tyrosine kinase signaling through the Shb adaptor protein regulates endothelial cell apoptosis.. Blood.

[31] Rehn M, Veikkola T, Kukk-Valdre E, Nakamura H, Ilmonen M (2001). Interaction of endostatin with integrins implicated in angiogenesis.. Proc Natl Acad Sci U S A.

