# Supplementary material for: A 3′ UTR SNP in COL18A1 Is Associated with Susceptibility to HBV Related Hepatocellular Carcinoma in Chinese: Three Independent Case-Control Studies
Source: PLoS One. 2012 Mar 26;7(3):e33855. doi: 10.1371/journal.pone.0033855 (PMC3312886; doi:10.1371/journal.pone.0033855)
Supplement: Table S1 — Multivariate Analysis for the Clinical Variables. (DOC) [file pone.0033855.s001.doc]

Table S1. Multivariate Analysis for the Clinical Variables

|  | 302_Beijing | | | Youan_Beijing | | |
| --- | --- | --- | --- | --- | --- | --- |
|  | P | OR | 95% CI | P | OR | 95% CI |
| Sex | .943 | 0.971 | 0.431-2.185 | .696 | 0.853 | 0.385-1.892 |
| Age | .017 | 1.032 | 1.006-1.058 | <.001 | 0.929 | 0.899-0.960 |
| Smoke | .005 | 0.373 | 0.188-0.739 | .067 | 0.500 | 0.239-1.049 |
| Drink | .314 | 0.703 | 0.353-1.397 | .001 | 0.291 | 0.145-0.583 |
| Family History | .215 | 1.465 | 0.801-2.677 | .001 | 2.811 | 1.491-5.301 |
| Hbeag | .061 | 1.809 | 0.973-3.365 | <.001 | 3.995 | 2.080-7.672 |
| Alt | <.001 | 1.015 | 1.009-1.020 | .001 | 1.014 | 1.006-1.022 |
| Ast | <.001 | 0.985 | 0.980-0.991 | .019 | 0.988 | 0.978-0.998 |
| Tbil | .001 | 0.929 | 0.890-0.970 | .285 | 0.969 | 0.913-1.027 |
| Dbil | <.001 | 1.112 | 1.049-1.178 | .764 | 0.980 | 0.860-1.117 |
| Log Hbvdna | <.001 | 1.409 | 1.245-1.595 | .748 | 0.978 | 0.854-1.120 |
