# Supplementary material for: A 3′ UTR SNP in COL18A1 Is Associated with Susceptibility to HBV Related Hepatocellular Carcinoma in Chinese: Three Independent Case-Control Studies
Source: PLoS One. 2012 Mar 26;7(3):e33855. doi: 10.1371/journal.pone.0033855 (PMC3312886; doi:10.1371/journal.pone.0033855)
Supplement: Table S2 — Primers and Probes Used in TaqMan Genotyping and mRNA Quantification. (DOC) [file pone.0033855.s002.doc]

Table S2. Primers and Probes Used in TaqMan Genotyping and mRNA Quantification

| Primers and probes | Sequence (5’ – 3’) |
| --- | --- |
| TaqMan genotyping |  |
| rs2183589 |  |
| Forward primer of PCR | TCACCATCTGTGATTCCAATTG |
| Reverse primer of PCR | CCAAATGGAAAACCAAAGAGATA |
| FAM labeled C allele | TTGTAATCCCTcGGAAACACGCT |
| HEX labeled T allele | TTGTAATCCCTtGGAAACACGCTC |
| rs2230688 |  |
| Forward primer of PCR | CGGCCCTAAAACCCAGG |
| Reverse primer of PCR | CCGTGGTCTGCTCCTCG |
| FAM labeled G allele | TCACCACgCCACCCTTGG |
| HEX labeled T allele | CACCACtCCACCCTTGGCTG |
| rs11702425 |  |
| Forward primer of PCR | CTGTGTCTCCTCTGCCCTGA |
| Reverse primer of PCR | GCCAGCAACCACAGAAACC |
| FAM labeled T allele | CCTGCCGGGACTtAAGGTCAGT |
| HEX labeled C allele | CTGCCGGGACTcAAGGTCAGT |
| rs7499 |  |
| Forward primer of PCR | GAGCCGCCGGTCCTCT |
| Reverse primer of PCR | GAGTGCCGCGAGCTGC |
| FAM labeled T allele | CCGGCCATCtGCATCCAGG |
| HEX labeled C allele | CCGGCCATCcGCATCCAG |
| mRNA quantification |  |
| COL18A1 |  |
| Forward primer of PCR | CTGTACAGCATCGTGCGC |
| Reverse primer of PCR | CTTCAGCGGACCCTCAGA |
| FAM labeled COL18A1 mRNA probe | ACGAGCTGCTGTTTCCCAGC |
| GAPDH |  |
| Forward primer of PCR | TCTGACTTCAACAGCGACAC |
| Reverse primer of PCR | CAAATTCGTTGTCATACCAG |
| HEX labeled GAPDH mRNA probe | CACTCCTCCACCTTTGACGCT |
